# X-ray Photoelectron Spectroscopy (XPS) Depth Profiling for Evaluation of La_2_Zr_2_O_7_ Buffer Layer Capacity

**DOI:** 10.3390/ma5030364

**Published:** 2012-02-27

**Authors:** Vyshnavi Narayanan, Klaartje de Buysser, Els Bruneel, Isabel van Driessche

**Affiliations:** Sol-Gel Centre for Research on Inorganic Powders and Thin Film Synthesis SCRiPTS, Department of Inorganic and Physical Chemistry, Ghent University, Krijgslaan 281-S3, Gent B-9000, Belgium; E-Mails: vyshnavi.narayanan@ugent.be (V.N.); els.bruneel@ugent.be (E.B.); isabel.vandriessche@ugent.be (I.D.)

**Keywords:** coated conductor, chemical solution deposition, buffer layers, depth profile, X-ray photoelectron spectroscopy

## Abstract

Lanthanum zirconate (LZO) films from water-based precursors were deposited on Ni-5%W tape by chemical solution deposition. The buffer capacity of these layers includes the prevention of Ni oxidation of the substrate and Ni penetration towards the YBCO film which is detrimental for the superconducting properties. X-ray Photoelectron Spectroscopy depth profiling was used to study the barrier efficiency before and after an additional oxygen annealing step, which simulates the thermal treatment for YBCO thin film synthesis. Measurements revealed that the thermal treatment in presence of oxygen could severely increase Ni diffusion. Nonetheless it was shown that from the water-based precursors’ buffer layers with sufficient barrier capacity towards Ni penetration could be synthesized if the layers meet a certain critical thickness and density.

## 1. Introduction

In a coated conductor architecture, c-axis oriented YBCO films for superconducting applications, must be deposited on flexible, **R**olling **A**ssisted **Bi**axially **T**extured (RABiTS) Ni-5%W substrates [1,2]. Ceramic buffer layers are deposited in between the substrate and YBCO layer to prevent chemical interactions and diffusion of the metallic substrate into the superconducting YBCO. The high temperature processing of YBCO layers in oxygen atmosphere can cause penetration of the Ni atoms from the substrate into the superconducting layer and oxidation of the Ni-5%W substrate. These adverse interactions need to be prevented by the presence of buffer layers which necessarily not only stop the diffusion but should also transfer the epitaxial texture of the RABiTS Ni-5%W substrate to the superconducting layer. Many buffer layers including CeO_2_, YSZ, La_2_Zr_2_O_7_, Gd_2_Zr_2_O_7_ have been prevalently used [3,4,5,6,7,8,9,10,11,12]. La_2_Zr_2_O_7_ (LZO) is used as one of the prominent buffer layer because of its closed packed pyrochlore structure with a lattice parameter of 10.79 Å. It has a low lattice mismatch to both YBCO and Ni-5%W substrate (0.5% and 1.8% mismatch to YBCO, a- and b-axes, respectively and 7.6% to RABiTS Ni-5%W substrate) [7]. Many different kinds of deposition techniques can be adopted for the deposition of the buffer layers and the final superconducting layer. Chemical solution deposition (CSD) has proven to be an important and cost-effective method for depositing YBa_2_Cu_3_O_7−x_ (YBCO) based second generation superconductors [13,14]. Furthermore, CSD is one of the cheapest and easily adaptable methods to suit the needs of the buffer layer deposition under non-vacuum conditions. Many of the CSD methods use methoxyethanol or propionic acid based solvents to prepare good quality solutions and in turn buffer layers of good texture and crystallinity [4,5,8,9]. However, water based LZO solutions are preferred for environmental and economic reasons. 

In our laboratory, LZO precursor solutions using water as main solvent have been prepared and buffer layers based on these solutions were deposited taking into account the study of Caroff *et al.* which reported a minimum thickness of 150 nm to ensure a sufficient texture quality and to absorb most of the defects of the substrate [15]. The effective use of these crack-free and crystalline LZO buffer layers of 40–150 nm thickness from water based solutions in transferring the texture to the superconducting layer was studied by depositing YBCO and measuring their critical current [9,10,16]. 

Besides extensive crystallinity studies published elsewhere, we here present an XPS study used as an effective tool in optimization of the performance of the LZO layer by studying the in-depth composition of the elements and the inter-diffusion thickness of the Ni substrate and the LZO layer [17]. These LZO layers were prepared under reducing conditions to prevent oxidation of the Ni-5%W substrate. Technically, the LZO buffer layers annealed under reducing conditions (in Ar-5%H_2_ gas) are further processed by depositing YBCO on top of them, under oxidizing conditions. The effective action of the buffer layer is to prevent the oxidation of the Ni-5%W substrate and penetration of Ni into the YBCO layer during the YBCO annealing under oxidizing conditions. There are reports that indicate that nickel oxide (NiO) is formed during the annealing step on the interface of the substrate and LZO layer after deposition of YBCO [16]. Here, the as-prepared LZO buffer layers from water-based precursors are processed under YBCO processing conditions (in oxygen atmosphere) without depositing YBCO. Their buffer layer action is compared with that of the as-prepared LZO buffer layers prepared under reducing conditions using XPS. Four different LZO layers from different water-based precursors under as-prepared and under YBCO annealing conditions were studied using XPS.

## 2. Results and Discussion

**Figure 1:**
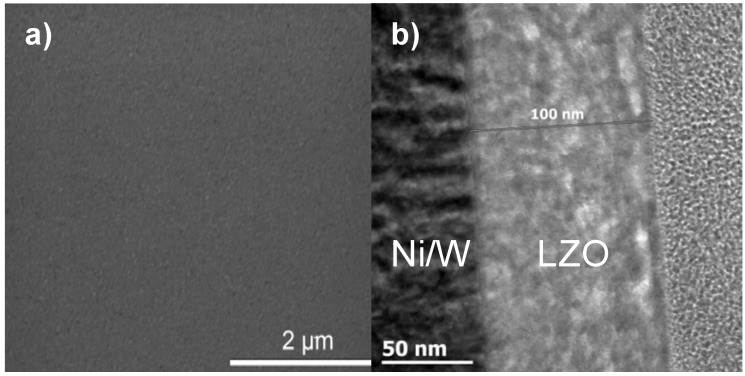
(**a**) Scanning and (**b**) Transmission Electronic Microscopy images of a thin layer prepared from system 2.

All layers exhibit crack free surfaces, unless stated otherwise, as is shown in Figure 1a, b for a layer synthesized using system 2. A TEM bright field image of a cross section throughout the LZO layer is shown in Figure 1b. Bright areas in the LZO layers are allocated to nanovoids [18]. The sample thickness of the different LZO layers is given in Table 1. The values for the thickness determined by ellipsometry accord well with the results from TEM measurements.

**Table 1:** Overview of the chemical composition of the precursors used for preparation of the Lanthanum zirconate (LZO) layers.

	Precursor salts	Chelating agent	Additives	Polymer	Thickness
System 1	La-acetate Zr-acetate hydroxide	TEA	Acetic Acid, TEA, NH_4_OH	-	40 nm ^a^
System 2	La-acetate Zr-n-propoxide	EDTA	Acetic Acid, EDA, EG NH_4_OH	-	110 nm ^a^ 100 nm ^b^
System 3	La-acetate Zr-n-propoxide	EDTA	Acetic Acid, EDA, EG NH_4_OH	PVP	220 nm ^a^ 205–225 nm ^b^
System 4	La-acetate Zr-n-propoxide	EDTA	Acetic Acid, AMP, NH_4_OH	PVP	125 nm ^a^ 150 nm ^b^

^a^ Thicknesses are measured by ellipsometry; ^b^ Thicknesses are measured by TEM.

**Figure 2:**
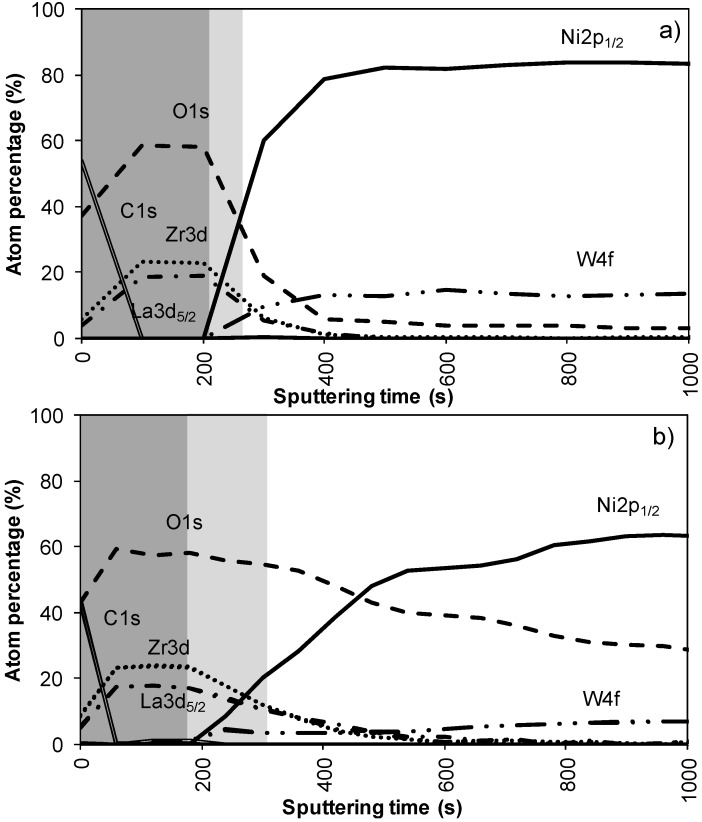
XPS depth profile of a buffer layered sample using system 1 (**a**) before and (**b**) after annealing.

Figure 2a,b shows the results of the XPS depth profiling for samples synthesized using system 1 before and after annealing respectively. From these figures it is observed that on top of the sample mainly carbon is present due to a thin layer of surface contamination. After one sputter cycle the majority of the carbon has been sputtered off and predominantly La, Zr and O peaks are registered. Results from XPS measurements are shown in Table 2.

The observed stoichiometry of La/Zr in the XPS measurements is not exactly 1:1. The use of standards for the determination of correction factors could compensate for this apparent off stoichiometry [17]. However there is no physicochemical reason to doubt the constitution of the LZO layers, thus no additional calibrations were performed. Nevertheless for each sample the presence of phase-pure La_2_Zr_2_O_7_ was confirmed with XRD/RHEED analysis. 

The thickness of the LZO layer is marked with a (light + dark) grey box on the graphs. The time it takes for the Zr and La concentration to diminish to half of their maximum value was taken as a limit. This approximately coincides with the point where the nickel concentration has increased until half its maximum value. The nickel-free zone is marked with a dark grey box. 

The sputter rate of La_2_Zr_2_O_7_ was determined by comparing the thickness of the film measured with TEM or ellipsometry with the time needed to sputter through the La_2_Zr_2_O_7_ layer, more precise the time it takes for the Zr and La concentrations to diminish to half their maximum value. In system 1, Figure 2a, this takes approximately 250 s, for a 40 nm thick LZO film inferring a sputter rate of 0.16 nm/s. Performed for all samples, the estimated average sputter rate for LZO is 0.17 ± 0.1 nm/s, or 1.13 times faster compared to the sputter rate of Ta_2_O_5_. This average sputter rate is used in this paper to correlate sputter time to thickness of specific zones. 

In system 1, Ni 2p^1/2^ and W 4f peaks were first observed after 300 seconds of sputtering. Based on the sputter rate of La_2_Zr_2_O_7_ it can be concluded that the nickel free region, marked as a dark grey area, on top of the sample is around 34 nm (200 s). After more than 500 s of sputtering the Zr and La peaks disappeared from the spectra. 

Annealing this sample, thus mimicking an YBCO deposition, resulted in an increase in diffusion of all elements (Figure 2b). The steepness of the curves vastly decreases and the region where both Ni/W and La/Zr are present is at least doubled. The thickness of the nickel free zone slightly decreased. Nickel is now observed after 240 s of sputtering, rendering a rather thin nickel free zone at the top of the sample.

**Table 2:** Overview of the XPS depth profiling experiments.

LZO layers	d_layer_ (elllipso-metry) (nm)	Strong increase in Ni signal after (s sputtering)	Zr signal at Half Max after (s sputtering)	Ni signal Start plateau (s sputtering)	First Ni peak (s sputtering)
System 1	40	200	250	400	300
System 1 YBCO annealed		180	300	550	240
System 2	~110	560	660	720	600
System 2 YBCO annealed		500	630	1200	550
System 3	280	810 Start 550 s Strong increase 1000	1600	2000	720
System 3 YBCO annealed		540 Start 260 s Strong increase 700	1100	1600	540
System 4	125–150	500	580	900	600
System 4 YBCO Annealed		500	670	1400	550

**Figure 3:**
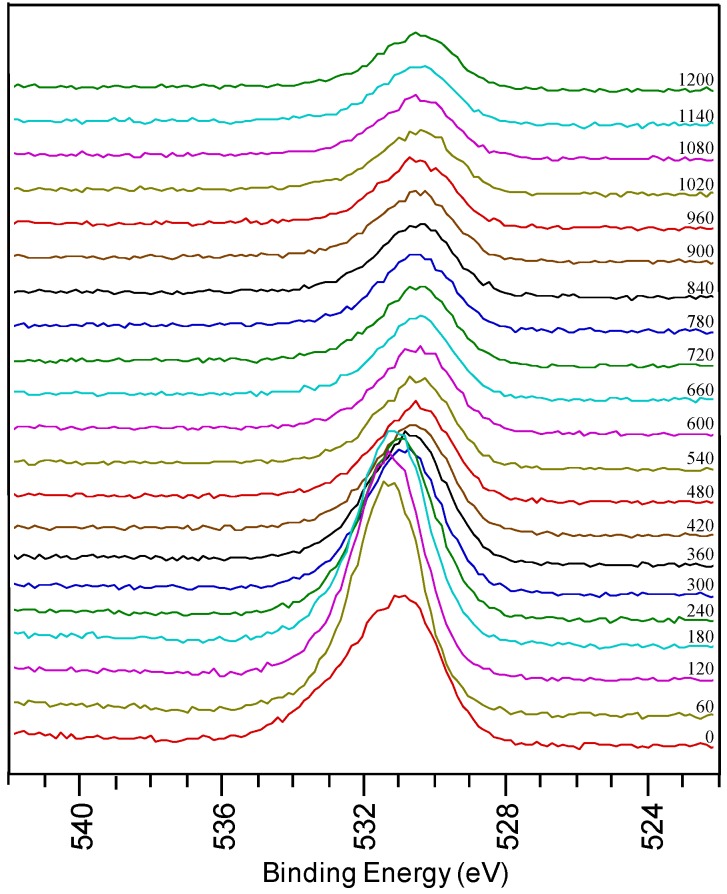
O1s region from a sample using system 1 after annealing.

In the detailed O 1s spectra measured on a thin film prepared by system 1 after annealing, a broad oxygen peak is observed on the surface of the sample. It can be deconvoluted into a peak at low binding energy assigned to surface contamination and a peak at high binding energy corresponding to LZO. There is a shift in the oxygen peaks at different depths of the sample, corresponding to the various oxides present. With sputtering, the intensity of the peak at 531.5 eV decreases when La and Zr concentrations diminish. It shifts towards 530.5 eV indicating that oxidized Ni is present in the inner part of the sample. During annealing the physical property of the sample also changed from shiny texture (as-prepared LZO layer) to a hazy, and mildly peeled off layer (under YBCO conditions). From the high percentage of oxygen in the inner part of the sample and the shift in oxygen peak position it can be concluded that during the annealing process the substrate is severely oxidized, confirming the observations made by Cloet *et al.* by TEM [16]. 

**Figure 4:**
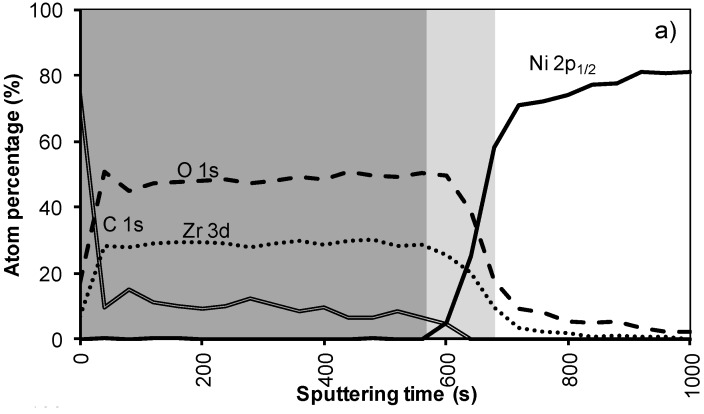

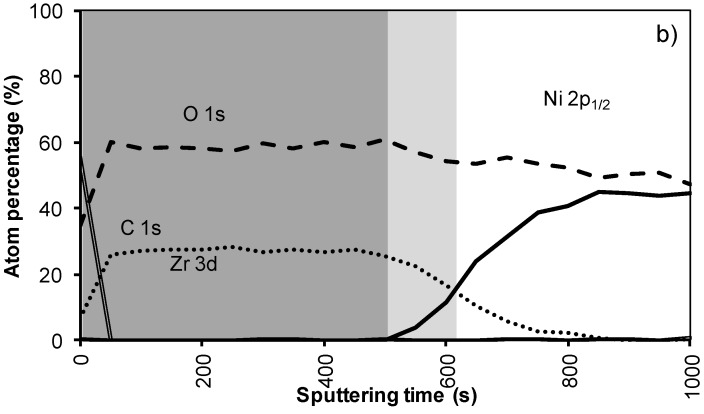
XPS depth profile of a buffer layered sample using system 2 (**a**) before and (**b**) after annealing.

Figure 4a shows the XPS depth profile of the sample prepared with system 2 before annealing. The increased viscosity resulted in a thicker oxide film. As could be expected from ellipsometry and TEM measurements, the LZO film and thus the nickel free zone is now much thicker. The first nickel peak appears in the spectrum after 560 seconds of sputtering. Another 100 sputter s later the concentration of Zr drop towards half the maximal value. The thickness of the LZO layer is confirmed to be approximately 110 nm thick. The spectra are free of Nickel during the first 560 s*.*

After annealing this sample shown in Figure 4b, again, the diffusion of Zr into Ni and vice versa vastly increased. The position at which the nickel concentration steeply increased is reduced from 100 to 85 nm (560 to 500 s respectively). In the mean-time the carbon content inside the layer disappeared. Presumably it was oxidized by the penetrating oxygen.

**Figure 5:**
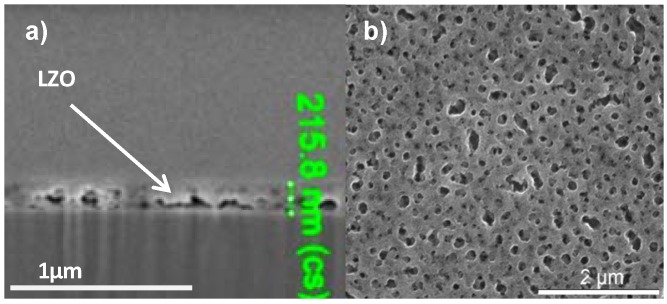
**(a)** Cross-sectional FIB-SEM micrograph; **(b)** SEM micrograph of the top surface of a sample prepared by system 3 before annealing.

In order to increase the critical thickness of the layers and the final nickel free zone after annealing, polyvinyl pyrrolidone (PVP) was added to the sol. System 3 is similar to system 2 but the addition of PVP increased the viscosity of the sol to 5.7 mPa.s and the layer thickness after dipcoating is at least doubled. Ellipsometry shows a layer thickness of 280 nm while FIB-SEM (Figure 5a) showed local thicknesses of 205–225 nm. Unfortunately, during the thermal process gases get trapped into the solution-gel system and the resulting layer exhibits a high porosity (Figure 5b) compared with Figure 1a. Those pores were present throughout the entire layer.

**Figure 6:**
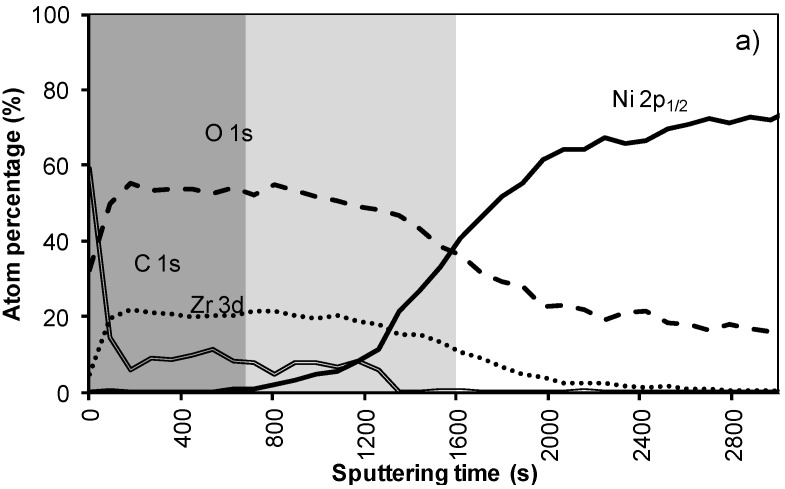

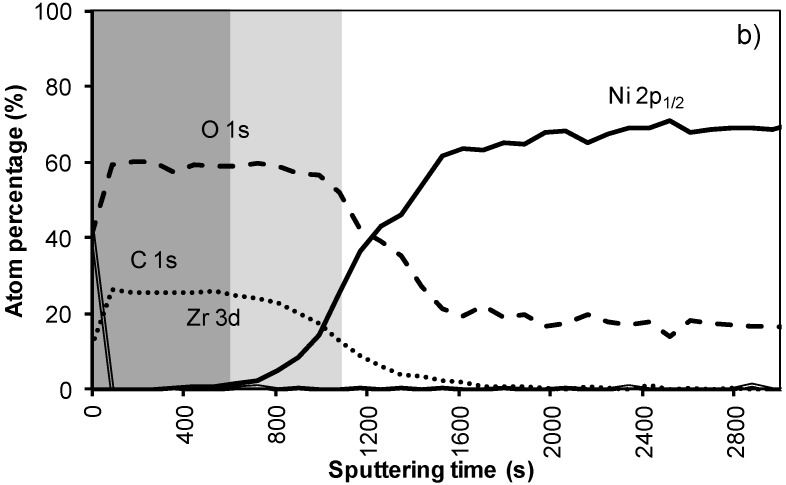
XPS depth profile of a buffer layered sample using system 3 **(a)** before and **(b)** after annealing.

In the XPS depth profiling (Figure 6a,b) of samples prepared by system 3, the first nickel peaks are observed after 120 nm (720 s). During annealing the nickel containing zone shifts with at least 30 nm (180 s). The increased thickness of the Ni free zone is related to the thicker LZO layer but the poor morphology causes an important reduction of the buffer capacity by 30% after annealing. A more dense but still thick enough LZO layer could lower the Ni penetration after annealing.

**Figure 7:**
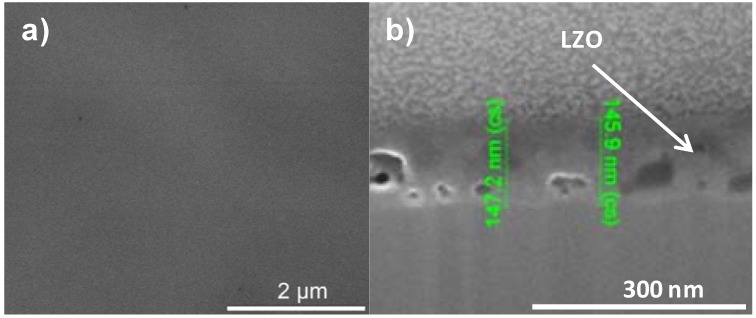
SEM micrograph of the (**a**) top surface and (**b**) cross section of a sample prepared by system 4 before annealing.

A dense, crack free film, with an intermediate thickness could be synthesized by using PVP but replacing ethylene diamine and ethylene glycol by AMP. This results in a precursor solution (system 4) with an intermediate viscosity of and a resultant layer thickness between 125 and 150 nm. SEM and FIB-SEM analysis on this sample confirmed that gases now could escape from the gel and a dense, crack-free top surface of the film was created as can be seen in Figure 7.

**Figure 8:**
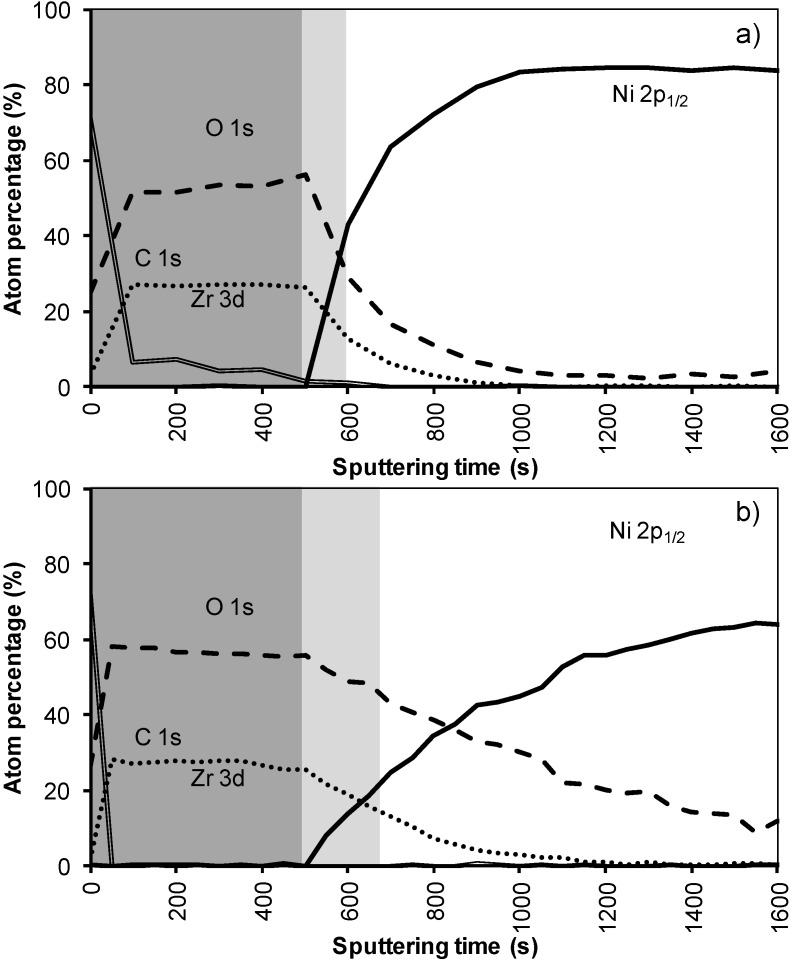
XPS depth profile of a buffer layered sample using system 4 **(a)** before and **(b)** after annealing.

The XPS depth profile of a film synthesized using this sol gel system 4 is shown in Figure 8. Before annealing the nickel free zone is estimated to be 85 nm (500 s) which is in the same order as system 2. As in all previous samples annealing results in an increase in oxygen content in the interior of the sample, pointing to the formation of NiO, removal of carbon and an increased interdiffusion of the adjacent layers. However, it is promising that with the increased layer thickness the nickel free zone of 85 nm could be maintained after annealing. Based on the above results, it can be summarized that the performance of the water based thin film (non-polymer and polymer included) against Ni penetration is strongly dependent on its thickness and morphology. But, layers from all these systems do not completely withstand the oxidizing condition under YBCO annealing completely as they are showing partial oxidation through the layers.

Additionally it has been shown that the film, prepared by system 4, is an appropriate buffer layer for the preparation of an YBCO coated conductor as a highly resembling film was used as a buffer layer for depositing an YBCO thin film by metal-organic decomposition (MOD) [19]. A buffer layer prepared by system 4 shows that CSD layers prepared by a well defined water-based chemistry can result in buffer layers preventing Ni diffusion and Ni oxidation.

## 3. Experimental Section 

### 3.1. Synthesis

#### 3.1.1. Precursor Solutions 

A detailed description of the different systems and explanation for the chosen chelating agents and polymers is given in previous publications [9,10,13]. The chemical composition is varied to improve the growth of crack-free LZO films with thicknesses ≥150 nm.

System 1 is a La acetate-Zr acetate-sol with TEA and acetic acid. The precursor solution was prepared using lanthanum acetate, La(CH_3_COO)_3_∙H_2_O, zirconium(IV) acetate hydroxide, Zr(CH_3_COO)(OH)_3_∙H_2_O, and acetic acid dissolved in water with triethanolamine (TEA) as a complexing agent. The acidity was set at pH = 7 using ammonia. The procedure for the following solutions was described in more detail previously [10]. In the final sol with a viscosity of 3.9 mPa.s the concentrations of the different metal ions were 0.17 M La^3+^; 0.17 M Zr^4+^; 0.67 M TEA and 3.3 M acetic acid. 

In system 2 a stoichiometric mixture of lanthanum acetate and zirconium n-propoxide (70% w/w in n-propanol) was diluted with acetic acid and water. A solution of ethylenediamine tetraacetate (EDTA) and ethylene diamine (EDA) was added to the metal salts solution. The pH of the mixed solution was adjusted to 6 by adding ammonia. Finally ethylene glycol (EG) was added to this solution at 60 °C, in order to increase the wettability and the viscosity of the solution. This solution was stirred and an excess of solvent was evaporated until concentrations of 0.2 M La^3+^; 0.2 M Zr^4+^; 0.8 M glycol; 1.6 M EDA; 0.2 M EDTA and 4 M acetic acid were obtained. The final solution had a viscosity of 4 mPa.s. 

System 3 is a water based La acetate-Zr n-propoxide-sol with with addition of polyvinyl pyrrolidone (PVP). The preparation method is the same as in system 2 but prior to evaporation polyvinyl pyrrolidone (PVP, Alfa-Aesar, MW 8000) was added to the solution. PVP is known to promote stress relaxation while heating up the gel, which makes it possible to synthesize thicker and crack free films [20]. The final concentration of the solution reached 0.2 M La^3+^; 0.2 M Zr^4+^; 0.8 M glycol; 1.6 M EDA; 0.2 M EDTA; 4 M acetic acid and 0.5 M PVP(monomer). The viscosity of the solution was found to be 5.7 mPa.s. 

System 4 is a water based La acetate-Zr n-propoxide sol with polyvinylpyrrolidone in which ethylene glycol and EDA were replaced by 2-amino-2-methyl-1-propanol (AMP). The final concentration is 0.2 M La^3+^; 0.2 M Zr^4+^; 0.8 M AMP; 0.2 M EDTA; 4 M acetic acid and 0.5 M PVP(monomer). The viscosity of the solution was found to be 4.8 mPa.s.

All the above prepared solutions were filtered using a 0.2 µm filter prior to dip-coating and have proven to be stable for at least 4 months. An overview of the different precursor solutions is given in Table 1.

#### 3.1.2. Deposition and Heat Treatment

Ni-5%W tapes of 1 cm wide and 80 µm thick (Evico, GmbH) were cut into strips of 2.5 cm length prior to dip-coating. The withdrawal speed of the dip-coating process was 20 mm/min for deposition of system 1 and 60 mm/min for deposition of systems 2 till 4. The as-deposited layers were transformed into a gel by drying at 60 °C for one hour. These amorphous layers were subjected to a optimized heat treatment to convert them into the desired crystalline phase. First they were heated from room temperature to 450 °C (ramp 1 °C/min) for 30 minutes. Next, a 3 °C/min heating rate was applied from 450 °C until 900 °C (dwell time = 1 h). Finally, the films were heated up to 1050 °C at 10 °C/min with a dwell time of one hour. The samples were left to cool inside the furnace. Conditions for system 1 are somewhat less invasive: the film was heated towards 900 °C with a constant heating rate of 5 °C/min. After a restrained dwell time of 50 min, the temperature was rapidly increased to 1050 °C and again maintained constant for an isothermal heat treatment of 50 min. The sample was cooled down to room temperature at a rate of 10 °C/min. All thermal treatments were carried out in forming gas (Ar-5% H_2_) at a constant gas flow between 0.4 and 0.55 L/min. 

A duplicate of each sample was additionally annealed in a oxidizing gas flow of 200 ppm O_2_ in N_2_ to simulate the YBCO process. The temperature was increased from room temperature to 815 °C at a rate of 10 °C/min. After 150 min the temperature was decreased to 400 °C at a rate of 3 °C/min. During this cooling ramp, the atmosphere was switched to oxygen (industrial quality) at 525 °C. After an annealing step of 5 h, the samples were furnace-cooled to room temperature.

This annealing step is identical to the heat treatment suitable for synthesis of YBCO thin films from an aqueous solution [21]. 

### 3.2. Characterization

#### 3.2.1. Determination of the Thickness and Microstructure of the Buffer Layer

The thickness of the layers was analyzed by spectroscopic ellipsometry (J.A. Woollam Co), fitting the experimental curve to model for LZO films on Ni-5%W substrates (wavelength = 638.3 nm and refractive index = 1.998). The microstructure of the surface was analyzed with a scanning electron microscope (FEG-SEM, FEI). Thickness was verified using a C_s_ corrected transmission electron microscope (TEM, JEOL 2200 FS) after an *in-situ* lift-out procedure with FIB (FEI, Nova 2000).

#### 3.2.2. XPS Analysis for Evaluating Buffer Layer Capacity

The ability of the buffer layer to withstand migration of Ni towards the surface was evaluated by XPS depth profiling (S-probe, Surface Science Instruments, VG with a monochromatic Al Kα-source, 1,486.6 eV). The voltage and power of the source were kept constant at 10 KV, 200 W. Sputtering of an area of 3 × 3 mm^2^ for 40 s was performed with an Ar^+^ ion gun (4 keV) resulting in a sputter rate for Ta_2_O_5_ of 0.15 nm/s. After each consecutive sputter cycle an area of 250 × 1000 µm^2^ was analyzed with an hemispherical analyzer. Regions for O 1s, C 1s, Zr 3d, La 3d^5/2^, Ni 2p^1/2^ and W 4f peaks were registered with a resolution of 0.15 eV. Peak areas were converted into atomic concentrations with the software package CasaXPS (Casa Software Ltd., UK) using a Shirley background and Scoffield sensitivity factors. The results for La and W are omitted from most of the graphs as they follow the same trend as Zr and Ni respectively.

## 4. Conclusions

It is shown that XPS depth profiling is a versatile tool to analyze thin films towards their ability to act as a buffer layer. Several LZO thin films are synthesized and annealed. Depth profiles allow us to compare the degree of oxidation in the inner part of the samples, degree of diffusion and thus width of nickel-free zones on top of the samples. It is thus shown that the Ni diffusion is influenced by the microstructure of the LZO layers. Comparing these results with ellipsometry and TEM cross sections show that the LZO sputter rate is 1.13 times the sputter rate of Ta_2_O_5_ or 0.17 nm/s in these experiments

An aqueous sol gel synthesis for LZO layers was produced that resulted in buffer layers that have a reasonable capacity of protecting subsequent layers from Ni diffusion. As could be expected, thick and dense layers show the best buffer capacity. This was confirmed with a 125–150 nm thick LZO layers prepared by an AMP-PVP adapted precursor solution where it was possible to obtain a nickel free zone of ±85 nm.
